# A Network Pharmacology-Based Study on the Anti-Lung Cancer Effect of Dipsaci Radix

**DOI:** 10.1155/2020/7424061

**Published:** 2020-04-27

**Authors:** Jiayan Wu, Shengkun Hong, Xiankuan Xie, Wangmi Liu

**Affiliations:** ^1^Shanghai Tenth People's Hospital, Tongji University School of Medicine, Shanghai 200032, China; ^2^Quzhou People's Hospital, No. 2 Zhongloudi, Quzhou 324000, China; ^3^The Second Affiliated Hospital, College of Medicine, Zhejiang University, Hangzhou 310009, China; ^4^Department of Neurology, Shanghai Tenth People's Hospital Chongming Branch, Shanghai, China

## Abstract

**Objective:**

Dipsaci Radix (DR) has been used to treat fracture and osteoporosis. Recent reports have shown that myeloid cells from bone marrow can promote the proliferation of lung cancer. However, the action and mechanism of DR has not been well defined in lung cancer. The aim of the present study was to define molecular mechanisms of DR as a potential therapeutic approach to treat lung cancer.

**Methods:**

Active compounds of DR with oral bioavailability ≥30% and drug-likeness index ≥0.18 were obtained from the traditional Chinese medicine systems pharmacology database and analysis platform. The potential target genes of the active compounds and bone were identified by PharmMapper and GeneCards, respectively. The compound-target network and protein-protein interaction network were built by Cytoscape software and Search Tool for the Retrieval of Interacting Genes webserver, respectively. GO analysis and pathway enrichment analysis were performed using *R* software.

**Results:**

Our study demonstrated that DR had 6 active compounds, including gentisin, sitosterol, Sylvestroside III, 3,5-Di-O-caffeoylquinic acid, cauloside A, and japonine. There were 254 target genes related to these active compounds as well as to bone. SRC, AKT1, and GRB2 were the top 3 hub genes. Metabolisms and signaling pathways associated with these hub genes were significantly enriched.

**Conclusions:**

This study indicated that DR could exhibit the anti-lung cancer effect by affecting multiple targets and multiple pathways. It reflects the traditional Chinese medicine characterized by multicomponents and multitargets. DR could be considered as a candidate for clinical anticancer therapy by regulating bone physiological functions.

## 1. Introduction

Lung cancer is one of the leading causes of death among all malignancies, especially in the elderly patients [1]. In China, lung adenocarcinoma accounts for the most common type of lung cancers, followed by lung squamous cell carcinoma [2]. So far, many treatments, including surgery, drug, radiation, and biological therapies, have been adopted for lung cancer therapy. Among patients with advanced non-small-cell lung cancer (NSCLC), median overall survival has increased by only 1.5 months despite the advent of such new therapies [3]. Over the year of diagnosis, mean spending for lung cancer is higher compared to breast and prostate [4]. Therefore, lung cancer has become a worldwide public health problem with poor prognosis and high cost, which brings a heavy burden to society.

Bone marrow-derived myeloid cells are abundant in the tumor stroma of lung cancer, and they can promote cancer growth [5]. For example, myeloid-derived suppressor cells (MDSCs) is important for tumor-associated immunosuppressive function, which is related to the occurrence, metastasis, and survival of cancer. In fact, MDSCs are a heterogeneous population of cells, including myeloid progenitor cells, immature granulocytes, immature macrophages, and immature dendritic cells [6]. There is emerging evidence that disruption of the programmed cell death protein 1 pathway with immune checkpoint inhibitors induces the immune escape of cancers with the backing of MDSCs [7, 8]. Researchers have tried other strategies to treat lung cancer based on MDSCs, such as promotion of myeloid cell differentiation, inhibition of MDSCs expansion, elimination of MDSCs, attenuation of MDSCs function, and so on [9]. Recently, Camilla et al. reported that lung cancer could deploy osteoblastic cells in bones remotely even in the absence of local metastasis, which supply lung cancer with neutrophils to foster cancer progression [10].

**Figure 1:**
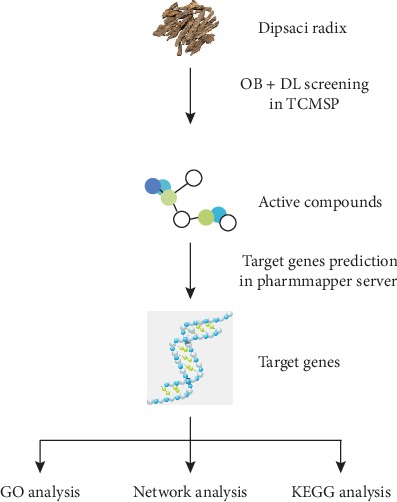
The flowchart of network pharmacology-based strategy for the anti-lung cancer effect of DR. Abbreviations: TCMSP: traditional Chinese medicine systems pharmacology database and analysis platform; GO: Gene Ontology; KEGG: Kyoto Encyclopedia of Genes and Genomes.

Dipsaci Radix (DR), also called Xu-Duan, is a traditional herbal medicine in China. According to the knowledge of traditional Chinese medicine, DR can be used for treating fracture because it can increase bone density and alter bone histomorphology [11]. The application of DR in the treatment of osteoporosis has been advocated because DR could decrease the loss of bone mass [12]. However, clinical applicability of DR in lung cancer remains to be determined. In view of the fact that both DR and lung cancer have effects on bone, DR may have a role in lung cancer treatment. Owing to this lack of related research, the aim of the present network pharmacology study was to define active compounds of DR, discover target genes of DR, and explore the potential mechanisms of DR in anti-lung cancer effect. The whole framework is shown in Figure 1.

## 2. Materials and Methods

### 2.1. Identification of Active Compounds

The ingredients of DR were obtained from traditional Chinese medicines systems pharmacology database and analysis platform (TCMSP, http://lsp.nwu.edu.cn/tcmsp.php). Oral bioavailability (OB) is an important indicator, which reflexes the extent to which oral drugs can overcome intestinal wall barriers to reach targets. Ingredients with lower OB may show a less beneficial therapeutic effect because fewer effective components enter the blood. The drug-likeness (DL) index was used to distinguish between drugs and nondrugs. Molecules with lower DL values mean that such molecules are less likely to be drugs [13]. In this study, the optimal cutoff values of OB and DL were 30% and 0.18, respectively. Ingredients of DR with values above these thresholds were considered as active compounds.

### 2.2. Target Genes Prediction

The interactions between each active compound and its target genes were obtained from PharmMapper server (http://www.lilab-ecust.cn/pharmmapper/). Using pharmacophore mapping approach, it can provide researchers with potential target candidates for the given probe small molecules [14, 15]. GeneCards, the human gene compendium, enabled us to effectively navigate and interrelate human genes and bone. Therefore, the target genes related to the anti-lung cancer effect of DR were considered as the overlap part of the results from the two above databases.

### 2.3. Network Construction

The complex relationships between lung cancer, DR, active compounds, and target genes were visualized using Cytoscape software (version 3.7.2) [16]. The Search Tool for the Retrieval of Interacting Genes (STRING) database [17] is a precomputed global resource, which is a powerful tool to explore and construct PPI network. In the present study, the PPI of the target genes was screened with a minimum required interaction score >0.95 using the STRING online tool (version 11.0). Target genes with high connectivity degrees in PPI network were recognized as hub genes.

### 2.4. Gene Ontology (GO) Term and Kyoto Encyclopedia of Genes and Genomes (KEGG) Pathway Enrichment Analyses

GO is a database for the unification of biology. It has three categories, including biological processes, molecular functions, and cellular components [18]. KEGG, a knowledge database, helps researchers to classify selected gene sets into their respective signaling pathways [19]. To explore the potential molecular mechanisms for the anti-lung cancer effect of DR, GO term and KEGG pathway enrichment analyses were performed using *R* software (version 3.6.1). *P* < 0.05 was set as the threshold value.

## 3. Results

### 3.1. Active Compounds Filtering

Search results from TCMSP showed that traditional ingredients of DR include 31 kinds of ingredients. Among these ingredients, total of 6 active compounds in DR with OB ≥ 30% and DL ≥ 0.18 were retrieved from TCMSP (Table 1).

### 3.2. Target Genes Prediction

**Figure 2:**
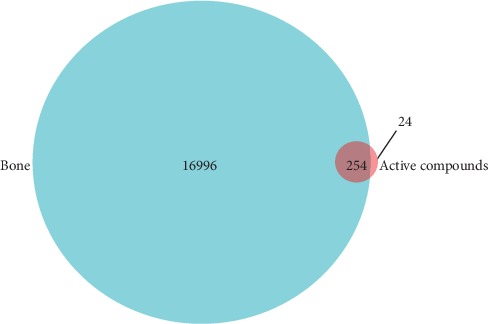
Venn diagram of the target genes for bone and active compounds. Bone has 17,250 target genes, while active compounds have 278 target genes. There are 254 overlapping target genes between the two sets.

17,250 target genes which could correspond to bone were identified from GeneCards (Table S1). Target genes with the normalized fit score were selected from PharmMapper server for every active compound. After duplicates elimination, 278 target genes for active compounds were identified according to the Uniprot identifiers mapping (Table S2). There were 254 overlapping target genes between bone and active compounds (Table S3 and Figure 2).

### 3.3. Protein-Protein Interaction (PPI) Network

**Figure 3:**
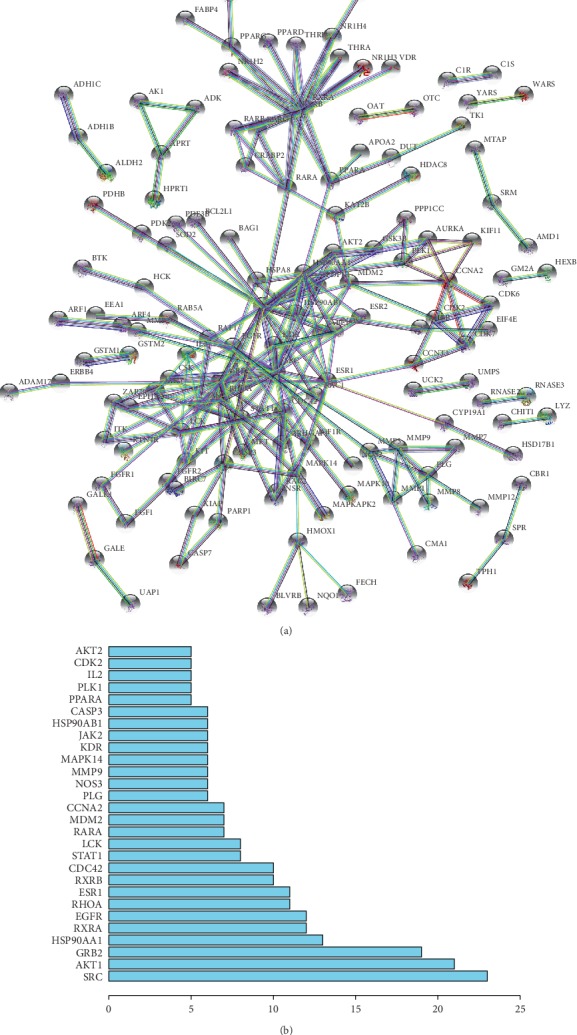
PPI network of the 254 overlapping target genes. (a) The PPI network plotting. (b) Number of connectivity degree for the hub genes. SRC has the highest connectivity degree.

PPI network of the 254 overlapping target genes was constructed using the STRING database (Figure 3(a)). Within a PPI network, each protein is presented by a node, and interactions are presented by the lines between nodes. The number of lines linked to a given node is defined as connectivity degree. Therefore, nodes that possess important biological functions usually have a high connectivity degree, which are also named hub genes. In this study, genes with a high connectivity degree (≥5) were defined as hub genes. SRC proto-oncogene (SRC), AKT serine/threonine kinase 1 (AKT1), and growth factor receptor bound protein 2 (GRB2) were the top 3 hub genes among the 28 hub genes (Figure 3(b)).

### 3.4. Network between DR, Active Compounds, Target Genes, and Lung Cancer

**Figure 4:**
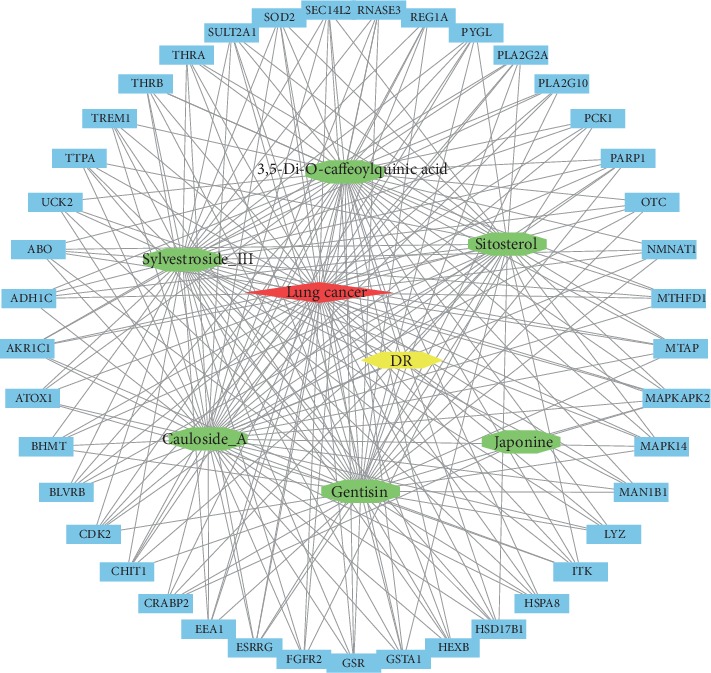
Network of the DR, active compounds, target genes, and lung cancer. Red diamond, lung cancer; yellow hexagon, DR; green octagon, active compounds; blue rectangle, target genes.

Target genes for at least 4 active compounds were included in the network construction (Figure 4). MAPK-activated protein kinase 2 (MAPKAPK2) was associated with all the active compounds in DR.

### 3.5. GO Term Enrichment Analysis

GO term enrichment analysis was categorized into three major terms as follows: biological processes, molecular functions, and cellular components. The top ten GO terms of each category are illustrated in Table 2. The most significantly enriched terms were significantly associated with steroid hormone-mediated signaling pathway, nuclear receptor activity, and vesicle lumen in the three categories, respectively.

### 3.6. KEGG Pathway Enrichment Analysis

**Figure 5:**
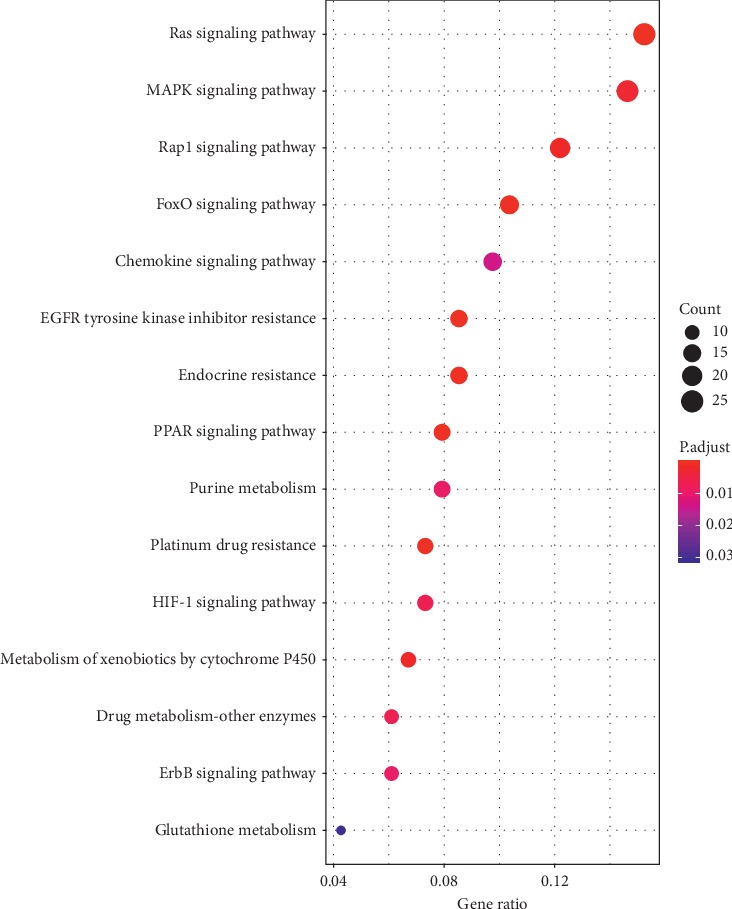
Bubble diagram of KEGG enrichment analysis. *Y*-axis label represents pathway and *X*-axis label represents gene ratio (gene ratio = amount of the genes enriched in the pathway/amount of all genes in background gene set). Size and color of the bubble represent the amount of genes enriched in pathway and enrichment significance, respectively. Ras signaling is the most enriched pathway.

The overlapping target genes were significantly associated with several signaling pathways and metabolisms (Figure 5).

## 4. Discussion

This study identifies systemic cross-talk between active compounds in DR and bone; these active compounds can modulate metabolism and physiology of bone. In turn, the host bone changes may inhibit lung cancer progression by impacting on hematopoiesis and osteogenesis.

There are six active components in DR. 5-Caffeoylquinic acid, a homology of 3,5-Di-O-caffeoylquinic acid, inhibits invasive activity of lung cancer cells mediated by the inhibition of p70(S6K)-dependent signaling pathway or the inactivation of Akt [20]. Moreover, caffeoylquinic acid derivatives, also known as strong antioxidant compounds, can effectively treat human leukemia and lung adenocarcinoma by inducing dysfunction of mitochondria [21]. Caffeoylquinic acid derivative also significantly decreased the proliferation level of bone marrow immature dendritic cells, whose function is often disrupted by cancer cells [22, 23]. Therefore, DR targeting bone may retard tumor growth and promote anticancer immunity. Sitosterol can ameliorate the state of bone fragility and fracture possibly due to estrogenic modulation [22]. In addition, several studies have indicated that sitosterol inhibit the proliferation of different cultured cancer cell lines such as prostate cancer, ovarian cancer, breast cancer, colon cancer, stomach cancer, leukemia, and lung cancer. The potential mechanism includes the stimulation of apoptotic cell death and the activation of cell cycle arrest [24–26]. Gentisin is found in Turpinia formosana Nakai, which possess robust osteogenic potential [27]. Bone metabolism is a homeostatic process, and its imbalance leads to the onset of diseases, including fostering cancer progression [10]. Therefore, gentisin deserves further research for development as antiosteoporotic agents. Japonine is a relaxant against rat's small intestine muscle, and its activity is similar to that of the typical muscle relaxant papaverine [28]. Sylvestroside III is naturally present in Tibetan medicine Pterocephalus hookeri, which could be used to treat low-grade chronic inflammatory synovitis and joint effusion [29]. Cauloside A shows anti-inflammatory efficacy [30]. No study exists for their role in bone or lung cancer.

Hub genes are highly interconnected with nodes in a module, which have been shown to have high biological relevance in a certain disease. Our study identified SRC, AKT1, and GRB2 as the top 3 hub genes. SRC is a proto-oncogene, which encodes a tyrosine-protein kinase. SRC regulates embryonic development and cell growth through phosphorylation by c-SRC kinase. SRC mutation is involved in the malignant progression of many types of cancers, including lung cancer. Ye et al. proposed that targeting SRC could be a strategy to overcome resistance to treatment based on epidermal growth factor receptor (EGFR) and tyrosine kinase inhibitors in aryl hydrocarbon receptor-activated NSCLC [31]. SRC inhibitors have been shown to reduce MDSCs in head and neck cancer and improve overall survival [32]. AKT1 is activated by platelet-derived growth factor. AKT1 phosphorylation can inactivate components of the apoptotic machinery in a transcription-independent manner. There are single disseminated tumor cells (DTC) in the bone marrow of patients with lung cancer. Akt1 regulates proliferation, survival, and migration in lung cancer-derived DTC [33]. Therefore, new bone-targeted agents targeting AKT1, such as DR, will show promise in lung cancer. GRB2 binds the EGFR and contains one SH2 domain and two SH3 domains, which are crucial for IL3 signaling in hematopoietic stem and progenitor cell. Conditional deletion of GRB2 induces a rapid decline of myeloid progenitors [34]. Myeloid cells modulate key cancer-associated activities and are associated with patient disease outcome [10, 35]. These findings indicate the potential anticancer activity of DR in lung cancer.

MAPKAPK2 were associated with all the six active compounds in DR, which encodes a member of the Ser/Thr protein kinase family. MAPKAPK2 can induce stress and inflammatory responses and regulates gene expression regulation and cell proliferation, which is controlled by direct phosphorylation through p38 MAP kinase. Hypoxia and serum depletion cause damage to cancer stem cells, which could be diminished by decrease in serine/threonine protein phosphatase 2A activity and activation of p38-MAPKAPK2-Hsp27 [36]. MAPKAPK2 chemical inhibition in bone marrow-derived macrophages impairs M2 macrophage polarization and M2 macrophage-induced angiogenesis [37]. Thus, interaction between DR and MAPKAPK2 may develop new strategies for lung cancer therapy.

In the present study, the most significantly enriched terms were steroid hormone-mediated signaling pathway, nuclear receptor activity, and vesicle lumen in the three GO categories, respectively. Cholesterol, a precursor to steroid hormone, is recently recognized to have important roles in regulation of normal and malignant hematopoiesis by regulating a wide variety of molecular machineries with its metabolites [38]. Nuclear factors and receptors, such as nuclear factor erythroid-derived 2 and retinoic acid receptor, play a vital role in hematopoietic stem and progenitor cell expansion and differentiation [39, 40]. Bone marrow contains some areas of the sinusoidal wall that are only composed of thin endothelial cells. There is no doubt that vesicle lumen is the most enriched cellular component.

Our study showed the enriched KEGG pathways were predominantly associated with signaling pathways. Ras signaling stimulates differentiation of osteoblasts from locally residing progenitor cells in a cell-autonomous fashion [41]. In turn, these osteoblasts supply tumors with tumor-promoting SiglecFhigh neutrophils, which foster cancer progression [10]. MAPK signaling pathway has been shown to be a part of differential gene expression profiles according to the transcriptome in bone marrow-derived cells from lung cancer [42]. These experimental data provide helpful information for bone-targeted therapy for lung cancer.

## 5. Conclusion

In summary, this study used a network pharmacology approach to construct a network to display the interactions between active compounds in DR and target genes. The results indicate that DR may exert anti-lung cancer effect by acting on bone at a molecular and systemic level. However, the results of network analysis prediction need verification via pharmacological and genetical methods. Given the involvement of DR in bone physiology, our study posits DR as a candidate drug for anti-lung cancer therapy.

## Figures and Tables

**Table 1:** Active compounds in DR.

ID	Molecule name	MW	OB (%)	DL
MOL003152	Gentisin	258.24	64.06	0.21
MOL008188	Japonine	295.36	44.11	0.25
MOL009322	Sylvestroside_III	686.83	48.02	0.53
MOL009312	3,5-Di-O-caffeoylquinic_acid	516.49	48.14	0.68
MOL000359	Sitosterol	414.79	36.91	0.75
MOL009317	Cauloside_A	444.72	43.32	0.81

MW, molecular weight; OB, oral bioavailability; DL, drug-likeness.

**Table 2:** The top 10 most significant terms in GO enrichment analyses.

Id		Description	*P* adjust	Count
*Biological processes*				
GO:0043401		Steroid hormone-mediated signaling pathway	8.30*E *−* *19	29
GO:0071383		Cellular response to steroid hormone stimulus	1.24*E *−* *18	32
GO:0048545		Response to steroid hormone	1.05*E *−* *17	37
GO:0009755		Hormone-mediated signaling pathway	3.41*E *−* *17	29
GO:0006367		Transcription initiation from RNA polymerase II promoter	5.08*E *−* *14	24
GO:0030522		Intracellular receptor signaling pathway	1.30*E *−* *13	28
GO:1901652		Response to peptide	2.44*E *−* *13	36
GO:0046777		Protein autophosphorylation	1.46*E *−* *12	24
GO:0072593		Reactive oxygen species metabolic process	5.02*E *−* *12	26
GO:0000302		Response to reactive oxygen species	9.15*E *−* *12	24

*Molecular functions*				
GO:0004879		Nuclear receptor activity	2.07*E *−* *24	21
GO:0098531		Transcription factor activity and direct ligand-regulated sequence-specific DNA binding	2.07*E *−* *24	21
GO:0003707		Steroid hormone receptor activity	2.07*E *−* *24	22
GO:0004713		Protein tyrosine kinase activity	5.32*E *−* *16	22
GO:0033293		Monocarboxylic acid binding	2.94*E *−* *10	13
GO:0031406		Carboxylic acid binding	1.41*E *−* *09	19
GO:0043177		Organic acid bindings	1.47*E *−* *09	19
GO:0004714		Transmembrane receptor protein tyrosine kinase activity	1.45*E *−* *07	10
GO:0004715		Nonmembrane spanning protein tyrosine kinase activity	1.52*E *−* *07	9
GO:0004175		Endopeptidase activity	1.52*E *−* *07	22

*Cellular components*				
GO:0031983		Vesicle lumen	8.41*E *−* *08	23
GO:0060205		Cytoplasmic vesicle lumen	1.35*E *−* *07	22
GO:0045121		Membrane raft	1.35*E *−* *07	21
GO:0098857		Membrane microdomain	1.35*E *−* *07	21
GO:0034774		Secretory granule lumen	1.69*E *−* *07	21
GO:0098589		Membrane region	1.69*E *−* *07	21
GO:0101002		Ficolin-1-rich granule	7.38*E *−* *05	13
GO:0031012		Extracellular matrix	2.70*E *−* *04	20
GO:1904813		Ficolin-1-rich granule lumen	2.70*E *−* *04	10
GO:0031256		Leading edge membrane	2.70*E *−* *04	11

## Data Availability

Data will be available upon request.

## Supplementary Materials

Supplementary Materials The following are available online. Table S1: target genes for bone. Table S2: 278 target genes for active compounds after duplicates elimination. Table S3: 254 overlapping target genes for active compounds and bone.
